# Supplementary material for: COZOID: contact zone identifier for visual analysis of protein-protein interactions
Source: BMC Bioinformatics. 2018 Apr 6;19:125. doi: 10.1186/s12859-018-2113-6 (PMC5889581; doi:10.1186/s12859-018-2113-6)
Supplement: Supplementary file 2 — Software build. Executable binary file of the software tool. (ZIP 118,784 kb) [file 12859_2018_2113_MOESM2_ESM.zip › cozoid/platform/docs/testng-6.5.1-javadoc.zip!/org/testng/ISuite.html]

ISuite (TestNG)


---


|  |  |  |
| --- | --- | --- |
| **Overview** \| **Package** \| **Class** \| **Tree** \| **Deprecated** \| **Index** \| **Help** | |  |
| **PREV CLASS**   **NEXT CLASS** | **FRAMES**    **NO FRAMES**     **All Classes** |  |
| SUMMARY: NESTED \| FIELD \| CONSTR \| METHOD | DETAIL: FIELD \| CONSTR \| METHOD |  |


---


## org.testng Interface ISuite

**All Superinterfaces:**: IAttributes, java.io.Serializable

**All Known Implementing Classes:**: SuiteRunner

---

``` public interface ISuite extends IAttributes ```

Interface defining a Test Suite.

**Author:**
:   Cedric Beust, Aug 6, 2004

---

| **Method Summary** | |
| --- | --- |
| `void` | `addListener(ITestNGListener listener)` |
| `java.util.List<IInvokedMethod>` | `getAllInvokedMethods()` |
| `java.util.List<ITestNGMethod>` | `getAllMethods()` |
| `org.testng.internal.annotations.IAnnotationFinder` | `getAnnotationFinder()` |
| `java.util.Collection<ITestNGMethod>` | `getExcludedMethods()` |
| `java.lang.String` | `getHost()` |
| `java.util.Collection<ITestNGMethod>` | `getInvokedMethods()`             **Deprecated.** *Use getAllInvokedMthods().* |
| `java.util.Map<java.lang.String,java.util.Collection<ITestNGMethod>>` | `getMethodsByGroups()`             Retrieves the map of groups and their associated test methods. |
| `java.lang.String` | `getName()` |
| `IObjectFactory` | `getObjectFactory()` |
| `IObjectFactory2` | `getObjectFactory2()` |
| `java.lang.String` | `getOutputDirectory()` |
| `java.lang.String` | `getParallel()` |
| `java.lang.String` | `getParameter(java.lang.String parameterName)` |
| `java.util.Map<java.lang.String,ISuiteResult>` | `getResults()` |
| `SuiteRunState` | `getSuiteState()`             Retrieves the shared state for a suite. |
| `XmlSuite` | `getXmlSuite()` |
| `void` | `run()`             Triggers the start of running tests included in the suite. |

**Methods inherited from interface org.testng.IAttributes**

`getAttribute, getAttributeNames, removeAttribute, setAttribute`

**Method Detail**

### getName

```
java.lang.String getName()
```

:   **Returns:**: the name of this suite.

---


### getResults

```
java.util.Map<java.lang.String,ISuiteResult> getResults()
```

:   **Returns:**: The results for this suite.

---


### getObjectFactory

```
IObjectFactory getObjectFactory()
```

:   **Returns:**: The object factory used to create all test instances.

---


### getObjectFactory2

```
IObjectFactory2 getObjectFactory2()
```

---


### getOutputDirectory

```
java.lang.String getOutputDirectory()
```

:   **Returns:**: The output directory used for the reports.

---


### getParallel

```
java.lang.String getParallel()
```

:   **Returns:**: true if the tests must be run in parallel.

---


### getParameter

```
java.lang.String getParameter(java.lang.String parameterName)
```

:   **Returns:**: The value of this parameter, or null if none was specified.

---


### getMethodsByGroups

```
java.util.Map<java.lang.String,java.util.Collection<ITestNGMethod>> getMethodsByGroups()
```

:   Retrieves the map of groups and their associated test methods.

    :   **Returns:**: A map where the key is the group and the value is a list of methods used by this group.

---


### getInvokedMethods

```
@Deprecated
java.util.Collection<ITestNGMethod> getInvokedMethods()
```

:   **Deprecated.** *Use getAllInvokedMthods().*

    :   Retrieves the list of all the methods that were invoked during this run.

        :   **Returns:**: a collection of ITestNGMethods belonging to all tests included in the suite.

---


### getAllInvokedMethods

```
java.util.List<IInvokedMethod> getAllInvokedMethods()
```

:   **Returns:**: a list of all the methods that were invoked in this suite.

---


### getExcludedMethods

```
java.util.Collection<ITestNGMethod> getExcludedMethods()
```

:   **Returns:**: All the methods that were not included in this test run.

---


### run

```
void run()
```

:   Triggers the start of running tests included in the suite.

---


### getHost

```
java.lang.String getHost()
```

:   **Returns:**: The host where this suite was run, or null if it was run locally. The returned string has the form: host:port

---


### getSuiteState

```
SuiteRunState getSuiteState()
```

:   Retrieves the shared state for a suite.

    :   **Returns:**: the share state of the current suite.

---


### getAnnotationFinder

```
org.testng.internal.annotations.IAnnotationFinder getAnnotationFinder()
```

:   **Returns:**: the annotation finder used for the specified type (JDK5 or javadoc)

---


### getXmlSuite

```
XmlSuite getXmlSuite()
```

:   **Returns:**: The representation of the current XML suite file.

---


### addListener

```
void addListener(ITestNGListener listener)
```

---


### getAllMethods

```
java.util.List<ITestNGMethod> getAllMethods()
```

:   **Returns:**: the total number of methods found in this suite. The presence of factories or data providers might cause the actual number of test methods run be bigger than this list.


---


|  |  |  |
| --- | --- | --- |
| **Overview** \| **Package** \| **Class** \| **Tree** \| **Deprecated** \| **Index** \| **Help** | |  |
| **PREV CLASS**   **NEXT CLASS** | **FRAMES**    **NO FRAMES**     **All Classes** |  |
| SUMMARY: NESTED \| FIELD \| CONSTR \| METHOD | DETAIL: FIELD \| CONSTR \| METHOD |  |


---
